# Training and transfer test to study the referential understanding of conspecific photographs by goats

**DOI:** 10.1007/s10071-025-01945-2

**Published:** 2025-03-13

**Authors:** Jan Langbein, Anja Eggert, Katrin Siebert

**Affiliations:** Research Institute for Farm Animal Biology (FBN), Dummerstorf, Germany

**Keywords:** Domestic ungulates, Familiarity, Picture equivalence, Representational insight, Visual discrimination

## Abstract

Individual recognition requires animals to compare available cues with stored information. For goats, living in stable social groups and forming social hierarchy, it is reasonable to assume they can discriminate between familiar and unfamiliar conspecifics. This study focuses on the cognitive mechanisms underlying goats’ perception of conspecific photographs, particularly whether they demonstrate image equivalence. Two groups of goats were trained to discriminate between portrait photographs of familiar and unfamiliar conspecifics. The goats in group A (*n* = 12) were trained to select familiar individuals, whereas the goats in group B (*n* = 12) were trained to select unfamiliar individuals. Subsequent transfer test was conducted to assess their ability to generalise learned preferences to novel photographs of previously unseen goats. During the first training tasks (Tr1 and Tr2), no differences in learning performance between the two groups were observed. However, in the later tasks (Tr3 and Tr4), the goats in Group A exhibited better learning performance than did those in Group B. In the transfer test, five goats in Group A, but only one goat in Group B, demonstrated preferences for novel familiar or unfamiliar conspecifics. The superior performance of Group A goats in Tr3 and Tr4 and the number of goats that successfully transferred the familiarity concept to novel individuals provide compelling evidence for the formation of true image equivalence. While goats can establish image equivalence through familiarity, the abstraction of unfamiliar concepts is a more challenging cognitive task.

**Supplementary Information:**

The online version contains supplementary material available at 10.1007/s10071-025-01945-2.

## Introduction

There is a famous painting by the Belgian surrealist Rene Magritte of a pipe with the words “Ceci n’est pas une pipe” underneath it, which means “This is not a pipe”. At first glance, since we can clearly see a pipe in the painting, this statement is slightly confusing. However, as the artist correctly pointed out, the painting is only a 2D representation of an arbitrary object. Our interpretation of it as a pipe is based on only our previous experience with photographs and paintings of a variety of similar objects. However, what about subjects who do not have any experience with visualisations of real objects or conspecifics? Humans who are not familiar with photographic representations of objects or conspecifics may not understand the referential meaning of their pictorial representations (Deregowski 2001). Only at approximately 2.5 years of age, after appropriate experience with pictorial representations, do children in Western societies begin to understand images as representations of reality (DeLoache 2004). This includes assigning specific characteristics to the depicted objects, which are stored together as mental copies. Zayan (1994) called this a single natural category (prototype).

The ability to discriminate 2D images of objects or conspecifics has been demonstrated for many different taxa, from insects to nonhuman primates (Bovet and Vauclair 2000). Recent studies have demonstrated advanced image discrimination capabilities for bees (*Apis mellifera*, Wu et al. 2013), geckos (*Correlophus ciliatus*, *Eublepharis macularius*, and *Phelsuma laticauda*, Katlein et al. 2022), keas (*Nestor notabilis*, O’Hara et al. 2015), giant pandas (*Ailuropoda melanoleuca*, Huang et al. 2023), and monkeys (*Cebus apella*, Truppa et al. 2009), as well as for various domestic animals, such as dogs (Range et al. 2008), pigs (Wondrak et al. 2018), cattle (Coulon et al. 2009), horses (Kappel et al. 2023), sheep (Bellegarde et al. 2017), goats (Nawroth et al. 2018), and chickens (Railton et al. 2014). However, the ability of an animal to discriminate between images does not tell us how the animal interprets the contents of these images. The animal may understand the images as visual stimuli without reference to the real objects shown, misinterpret the images themselves as real objects, or ultimately consider the images representations of real objects. Fagot defined these three cognitive approaches for processing 2D stimuli as (i) independence, (ii) confusion and (iii) equivalence (Fagot et al. 2010). Pictorially naive animals may confuse realistic images with their reference objects. The confusion (iii) mode can therefore be seen as the starting point in the dynamic system of picture processing. From there, Fagot describes (i) as the low road and (iii) as the high road of processing “repeatedly experienced pictorial stimuli depicting animate or inanimate objects encountered in life”. According to Brajon et al. (2015), the processing of 2D images at level (iii) is necessary for the individual recognition (IR) of conspecifics in images or photographs.

The understanding of the relationship between visual representations and real-world individuals by animals has been investigated in several experimental studies. The ability of an animal to recognise conspecifics by matching images to voices suggests the formation of internal representations of others. For example, a cross-modal expectancy violation paradigm was used to test whether large-billed crows (*Corvus macrorhynchos*) could identity congruence between visual representations of group members and subsequent playbacks of contact calls (Kondo et al. 2012). The findings suggest that crows spontaneously combine visual and auditory information from familiar conspecifics but not unfamiliar conspecifics. The same paradigm was used to show that penguins (Baciadonna et al. 2021), lions (Gilfillan et al. 2016) and goats (Pitcher et al. 2017) are capable of audio-visual cross-modal IR.

To distinguish between the simple ability to discriminate images of conspecifics as visual stimuli and test for some degree of class recognition or IR of conspecifics in images, other groups have used visual discrimination approaches related to the concept of familiarity. For example, Wilkinson (Wilkinson et al. 2010) trained pigeons to discriminate between photographs of familiar birds from the same aviary and photographs of unfamiliar birds. Two of the six photograph-naive pigeons learned the task. In another study, Stephan et al. (2013) trained pigeons to discriminate between photographs of familiar and unfamiliar conspecifics. Subsequent transfer experiments revealed that the pigeons could transfer previously learned categorical familiarity rules to novel, unseen images of other individuals of the same category. Moreover, Vonk and Hamilton (2014), who tested four orangutans and one gorilla for their understanding of photographs as depictions of real individuals, revealed that the animals could (1) match photographs at different time points in a delayed matching-to-sample test, presumably by identifying identical physical features, and (2) learn to select photographs of familiar individuals over photographs of unfamiliar individuals in a two-alternative test. Moreover, when novel pictures of both familiar and unfamiliar individuals were used, three of the animals were successful in a transfer test. In addition, Dasser (1987) investigated the concept of familiarity with three long-tailed macaques (*Macaca fascicularis*). The animals were first trained to discriminate familiar conspecifics on slides. Next, they successfully matched different views of the same subject. In a subsequent matching-to-sample (MTS) test, one macaque matched new slides of familiar and unfamiliar conspecifics to the corresponding categories presented in the 1st trial. These experiments indicate that monkeys can match photographs with real conspecifics and assign them to classes such as familiar and unfamiliar. Furthermore, Dittrich (1994) trained macaques (*Macaca fascicularis*) to discriminate between line drawings of different body parts of monkeys. The animals learned to discriminate and match different images of the same individual even when they differed in size and orientation. The results showed that the animals could generalise different views of facial stimuli (frontal or lateral). In two other studies (Pokorny and de Waal 2009a; Pokorny and Waal 2009b), the oddity paradigm, presented as a computerised 4-choice discrimination task, was used to test whether capuchin monkeys (*Cebus apella*) can discriminate the faces of familiar and unfamiliar conspecifics on the basis of identity. Initially, the animals were trained to discriminate one in-group member from three out-group members, or vice versa. In transfer tests, the animals applied the concept they had learned to both new pictures of the same subjects and pictures of juvenile conspecifics that had not previously been presented. All these studies show that different species can understand the referential relationship between drawings or photographs of conspecifics and real individuals at the class or even individual level through image equivalence (Pokorny and de Waal 2009b).

The ability to discriminate 2D images of conspecifics has also been successfully demonstrated for various domestic species, including chickens (Ryan and Lea 1994), horses (Ragonese et al. 2021) and pigs (Brajon et al. 2015; Wondrak et al. 2018). Sheep have been shown to discriminate images of conspecifics on the basis of facial cues (Kendrick et al. 1995); moreover, sheep discriminate faces of sheep faster than geometric patterns and faces of sheep of known breeds faster than faces of sheep of unknown breeds (Kendrick et al. 1996). Kendrick and colleagues interpreted these findings as prerequisites for the ability of the brain to store a mental prototype of the characteristics of another individual (Zayan 1994). Coulon et al. (2007) reported that heifers (*Bos taurus*) can discriminate 2D images of different breeds of cattle from other domestic species, that heifers can group different 2D views of the same familiar conspecific into a single category (Coulon et al. 2009), and that cattle can discriminate between facial views of familiar and unfamiliar conspecifics presented as 2D images (Coulon et al. 2011). We have used an automated learning device to present 4-choice visual discrimination tasks, showing that goats can discriminate between simple and complex visual stimuli (Langbein et al. 2006), improve their learning performance in successive similar tasks (Langbein et al. 2007a), and retain learned sets of visual stimuli for several weeks (Langbein et al. 2008). Moreover, goats were able to form categories on the basis of similarities in the visual appearances of artificial symbols and generalise this learned information to new symbols (Meyer et al. 2012).

In a recent study (Langbein et al. 2023), we trained goats to discriminate between photographs of familiar and unfamiliar conspecifics that were presented on a computer screen with a task design similar to that of Pokorny and Waal (2009b). The goats learned to discriminate familiar and unfamiliar individuals at the same rate and needed between 90 and 170 trials to reach the predefined learning criterion. In a subsequent reversal test, goats took more than twice as many trials to satisfy the learning criteria as they did during training. This result suggests that the animals learned to sort the photographs into ‘familiar’ and ‘unfamiliar’ categories during training and had to overcome this previously learned contingency in the reversal test before starting to learn the new rule. In the present study, we aimed to consolidate these findings on image equivalence in goats. We used a transfer test to determine whether goats spontaneously categorise unseen photographs of goats as either ‘familiar’ or ‘unfamiliar’ on the basis of the categories they had previously learned during training. The training protocol was similar to that used in our previous study.

## Animals, materials and methods

### Animals

The study was conducted between April and June 2022 at the goat experimental facility at the Research Institute for Farm Animal Biology (FBN, Dummerstorf, Germany). Most work on cognitive capacities of nonhuman animals has focused on humans’ closest relatives, i.e., primates in general and great apes in particular. Additionally, in terms of convergent cognitive evolution, research has included corvids and canids. However, compared to the amount of cognitive research that has been conducted on the aforementioned species, studies on the cognitive capabilities of other species are relatively underrepresented (Webster and Rutz 2020). Farm animals provide unique opportunities to study cognition in species that live in complex social groups or interact closely with humans. In this study, 24 female goat kids were included. The kids were weaned from their mothers at the age of 60 days and assigned to two equally sized experimental groups (A and B). The animals in the two groups had been kept in separate groups with their mothers until they were weaned and were therefore not visually familiar with each other. The pens for the two groups were in the same barn. They were of similar size (24 m2) and equipped identically (straw as bedding, a two-floor wooden climbing rack, concentrate provided twice daily (120 g/day/animal, Garvo bv. Holland, Nr. 5085), and hay available ad. lib.). Each pen had a separate compartment with opaque outer walls in which the learning device was installed, which could be entered by only one animal at a time. Drinking water was provided only as a reward at the learning device (Langbein et al. 2007b).

### The learning device

The design of the learning device is described in detail in Langbein et al. (Langbein et al. 2023). In summary, it consists of a 17″ touchscreen connected to a desktop computer. For identification at the learning device, the goats were fitted with an electronic transponder attached to a collar. The goats were trained with different 4-choice visual discrimination tasks presented via the touch screen, each consisting of one correct and three incorrect stimuli. The four stimuli were presented in sensitive fields of 7 × 7 cm. By pressing their nose on the screen, the goats could select a stimulus. The control software was used to manage the presentation of the stimuli on the screen, register the goats’ actions at the learning device and provide 30 ml of drinking water in a bowl under the screen as a reward for correct choices. In conjunction with the reward, two tone signals, which differed in correct and incorrect trials, were used as secondary acoustic reinforcers (Langbein et al. 2007b). The learning device was accessible 24/7. Each goat was free to choose when to visit the learning device and how many actions to perform per visit.

### Shaping

**Fig. 1:**
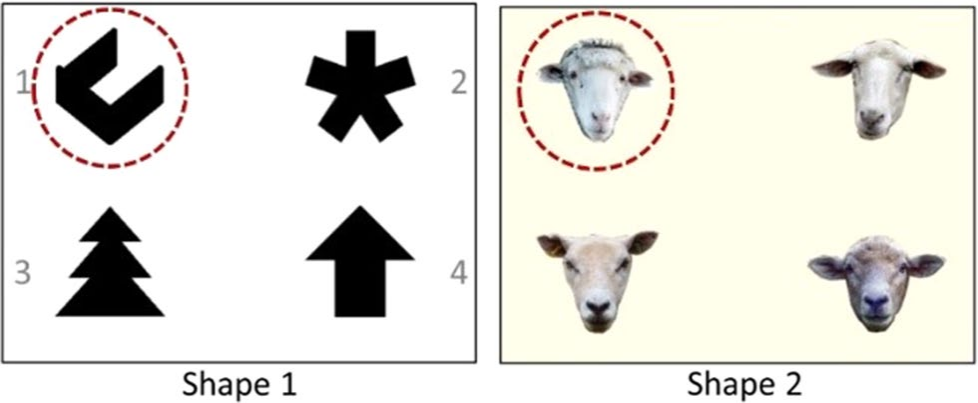
Visual four-choice discrimination tasks used in the last phase of shaping. The rewarded stimulus (S+) in each task is marked with a red circle here for identification purposes only

After being weaned from their mothers (mean age of 60 days), the goat kids in the two experimental groups were gradually shaped to the learning device. For three days, a float switch was hung in the water bowl to keep it half full, while a red dot (2 cm) was displayed on the screen directly above the switch. When the red dot was touched, an additional 30 ml of water was added to the bowl. Next, the float switch was removed. The goats had to touch the red dot for a 30 ml portion of water to be added to the bowl (four days). The red dot was then presented on the middle of the screen, 20 cm above the bowl (eight days). Finally, two red dots were placed 20 cm above the bowl (approximately 23 cm apart), which alternately activated the water dispenser every day (seven days). These phases in the shaping process took a total of 3 weeks to complete. As the final phase, the goats were presented with two consecutive 4-choice discrimination tasks via the touchscreen (Fig. 1).

The goats were trained for 14 days for each task. For the first task (Shape 1), we used four black shapes on a white screen, and for the second task (Shape 2), we used four portrait photographs of sheep faces presented on a uniform light yellow background (for details see Langbein et al. 2023). To receive a reward, the goats were required to select the stimulus that had been predetermined as S+ (see Fig. 1) by touching it with their nose. The arrangement of the stimuli in consecutive trials followed two pseudorandom sequences of 24 possible stimulus combinations to ensure that each of the four stimuli was evenly presented across the four positions on the screen. Each trial was followed by an intertrial interval of 3 s, in which a black screen was presented, before the stimuli were presented with a different arrangement in the next trial. The control software ensured that any tendency of the goats to establish side preferences was counteracted at all times (Langbein et al. 2006). By the end of the shaping phase, all the goats had associated the on-screen images with the water supply and could meet their daily water needs (approximately 1 L).

### Training

**Fig. 2:**
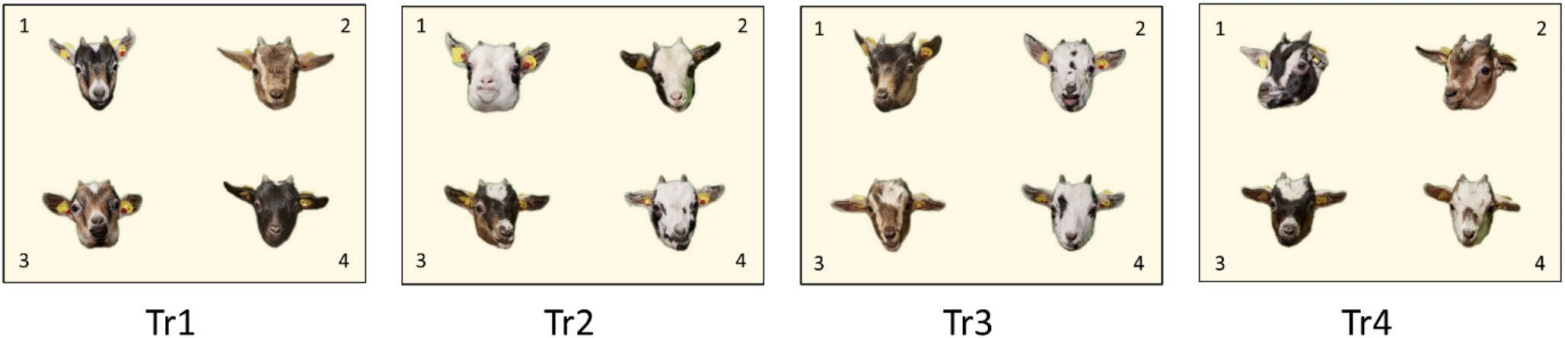
Four discrimination tasks (Tr1-Tr4) that were used during the training of experimental Groups A and B. The correct photograph was always of a Group A goat (here, the top left in each case). The other three photographs were of goats from Group B. The four photos in each task are numbered here for identification purposes only (P1-P4)

During training, both groups were presented with a series of four discrimination tasks (Tr1-Tr4). The photographs presented in each task were identical for both groups. In the tasks, a portrait photograph of an animal from Group A was the correct stimulus, and three photographs of animals from Group B were incorrect distractors (Fig. 2). Thus, Group A was trained to always choose a photograph of a member of their own group, whereas Group B always had to choose a photograph of an unfamiliar goat.

The arrangement of the four photographs in consecutive trials was similar to that in the two final tasks of the shaping phase. However, we used different pseudorandom sequences of the 24 possible stimulus combinations in each task. Additionally, we used six slightly different portrait photographs of each goat in each task. The size of the individual photographs on the screen was 7 × 7 cm. Each task lasted seven days. To determine the spontaneous preferences of the goats for individual photographs, we conducted one-day pretests (PT1-PT4) in which all choices were rewarded. For the next six training days, rewards were provided only if the correct photograph was chosen. For further training details, please refer to Langbein et al. (2023).

### Transfer test

**Fig. 3:**
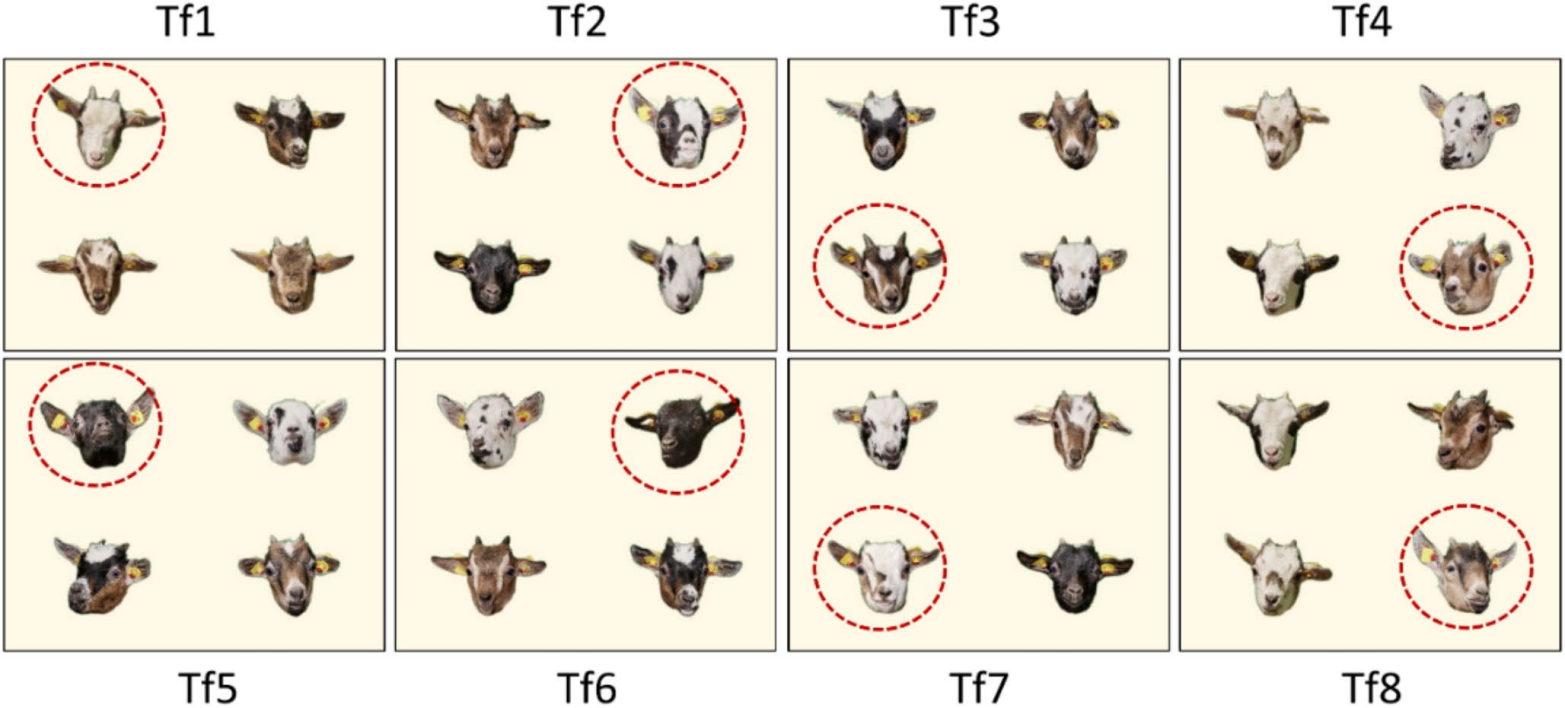
Eight new stimulus combinations (Tf1-Tf8) that replaced eight stimulus combinations in Tr4 in the transfer test. The stimulus combinations consisted of one photograph of a goat from Group A that had not been shown before (correct) and three photographs of goats from Group B that had been used before but were rearranged (incorrect). The correct photographs are marked with red circles here for identification purposes only

Upon successfully completing the four training tasks, a transfer test was conducted to prove that the goats had formed the categories “familiar” and “unfamiliar” during the training. For this transfer test, Tr4 continued to be presented. However, eight of the 24 stimulus combinations were replaced with new combinations (Tf1-Tf8). These new combinations consisted of one photograph of a previously unseen goat from Group A and three photographs of goats from Group B that were rearranged (Fig. 3).

### Data scoring

Since individual goats in the compartment with the learning device were undisturbed by the remaining goats in the group, the individual animals within the group were regarded as independent replicates for the statistical analysis. To prevent any form of social learning, the compartment containing the learning device was surrounded by opaque walls (Langbein et al. 2007b). Goats that were shown a photograph of themselves in any of the tasks, either as correct or incorrect, were excluded from the analysis for the task. We carried out PTs to assess the goats’ preferences for specific photos in different training tasks and analysed the preference score, i.e. percentage of choice (%choice) for each of the four photographs. In the training tasks (Tr1-Tr4), we analysed learning performance as daily success rate (%) in terms of percentage of correct choices and absolute learning performance by calculating the number of trials required for each goat to reach the learning criterion (TtC), defined as 46% correct choices in at least two consecutive sequences of 20 trials. (Hanggi 1999; Langbein et al. 2007a). We finally examined the number of correct choices of each animal in the transfer test.

### Statistical analysis

Statistical analyses were performed using R version 4.4.0 (R Core Team 2024) and the tidyverse R package version 2.0.0 (Wickham et al. 2019) was utilized for data processing and visualization.

We investigated whether belonging to one of the two groups (Group A, Group B) or the specific photograph (P1-P4, see Fig. 2) explained the variation in the spontaneous preference score (%choice) of the 24 female goat kids in the PTs. We fit linear models with g*roup* (2 levels) and *photo* (4 levels) and their interaction as fixed effects, separately for each PT (PT1-PT4). Owing to the violation of the homogeneity of variance assumption in PT3, we applied a Box‒Cox transformation, which successfully addressed this issue. Further diagnostic checks (normality of the residuals) revealed no violations of model assumptions. We conducted an ANOVA based on the fitted model, and we report the F statistics and p values of the fixed effects.

We also examined how success rate of the goats improved over six consecutive training days, i.e. when only choosing the correct photograph was rewarded. We investigated whether belonging to one of the two groups (Group A, Group B) or the specific training task (Tr1-Tr4) had an impact on the results. As the training task were performed by each animal on six consecutive days, we fitted a Generalized Least Squares (GLS) model using the gls function from the “nlme” package (Pinheiro et al. 2023) to examine the effects of *group* (2 levels) and *Tr *(4 levels) on learning success. Fixed effects included group, Tr, and their interactions. We accounted for a nested grouping structure of individual animals within groups and addressed serial correlation from six repeated measurements per animal (test day). Given the evenly spaced time points, we modeled the residual covariance using a first-order autoregressive AR(1) structure. To capture the nonlinear saturation of learning success over time, we incorporated natural cubic splines with two knots. Although learning performance was scored as daily success rate (%), the distribution of the data was not very skewed (the values were approximately 60% on average), and we fit the model based on the original scale. Further diagnostic checks (normality of the residuals) revealed no violations of model assumptions. We conducted an ANOVA based on the fitted model, and we report the chi-square statistics and p values of the fixed effects. The estimated marginal means (EMMs) were calculated via the emmeans function in the package “emmeans” (Lenth 2024), and comparisons between the groups were tested. For a better interpretation of the regression coefficients obtained via the model output, we plot the EMMs, which represent the average results along with the 95% confidence intervals for specific combinations of the predictor values.

To analyse the absolute learning performance in Tr1-Tr4, we fit a linear mixed model using the lmer function of the package “lmerTest” (Kuznetsova et al. 2017) with *group* (2 levels), *Tr* (4 levels) and the 2-way interactions as fixed effects. We also considered a random animal effect. The TtC values were log-transformed to meet the model assumption of normally distributed response variable and homogeneity of variances. Further diagnostic checks (normality of the residuals) revealed no violations of model assumptions. We conducted an ANOVA on the results of the model fit to get the F-statistic and p-values of the fixed effects.

To prove that the goats had formed the categories “familiar” and “unfamiliar” during training, success in the first presentation of each of the interspersed stimulus combinations in the transfer test was tested for each animal via a two-tailed binomial test.

### Ethical note

During shaping the kids were stepwise trained to interact with the touch screen of the learning device to get drinking water (see secion (c) *Shaping*). Drinking water was only delivered as a reward at the learning device throughout the course of the learning experiment. However, this in no way meant that water consumption was restricted when learning success was low, as the learning device was available 24 h a day and the dwarf goats could make as many trials at the learning device as they wanted. So the animals simply had to increase the number of trials to compensate for the low learning success, and so they did. To give an example, in an early study (Langbein et al. 2006), the individual number of trials increased up to 200 per day at the beginning of the first learning task. Postulating a learning performance of only 20%, this resulted in a delivery of 1.2 L of water, which is in the range of normal intake rate of the animals (Rossi and Scharrer 1992; Rossi et al. 1999). Nevertheless, to make sure that the goats had enough drinking water in all sections of the learning experiment, we surveyed daily water consumption based on the data from the learning device. We uploaded a figure for individual daily water consumption for the four discrimination tasks (Tr1-Tr4) as supplementary material. Daily water consumption ranged for most animals from 0.75 to 1.25 L and was relatively stable for individuals across the four tasks. We did not find any signs of serious distress or impaired weight gain in the dwarf goats at any time during the experiments.

All the experimental procedures were performed in accordance with the German “Tierschutz-Versuchstierverordnung” (Animal Protection Experimental Regulation). The Committee for Animal Use and Care of the Ministry of Agriculture, Environment and Consumer Protection of Mecklenburg-Western Pomerania, Germany, approved all the animal handling and treatment procedures (Ref. 7221.3-2-014/20).

## Results

### Pretest (PT)

The goats showed certain preferences for individual photographs in all PTs exept PT1 (see Table 1). Photographs 1 and 4 were the most preferred in PT2, photograph 2 was the most preferred in PT3, and photographs 1 and 3 were the most preferred in PT4 (Fig. 2). These preferences did not differ between the two groups. A single preference for the subsequent correct picture was not observed in any of the four PTs.

**Table 1:** Results of the 2-way ANOVA for the impact of *photo* and *group* on the preference score (%choice) in the PTs (PT1-PT4)

ANOVA PT1	Sum Sq	Df	F value	Pr (> F)	ANOVA PT3	Sum Sq	Df	F value	Pr (> F)
Group	0	1	0	1	Group	0.84	1	0.12	0.731
Photo	712.52	3	1.45	0.237	Photo	3095.85	3	147.53	<0.001
Group: Photo	1268.04	3	2.57	0.061	Group: Photo	8.77	3	0.42	0.741
Residuals	11828.61	72			Residuals	475.64	68		

ANOVA PT2	Sum Sq	Df	F value	Pr (> F)	ANOVA PT4	Sum Sq	Df	F value	Pr (> F)
Group	0	1	0	1	Group	0	1	0	1
Photo	20654.3	3	139.64	<0.001	Photo	19569.47	3	114.23	<0.001
Group: Photo	170.8	3	1.15	0.333	Group: Photo	103.62	3	0.6	0.614
Residuals	3549.77	72			Residuals	3883.12	68		

### Training (Tr1-Tr4)

**Fig. 4:**
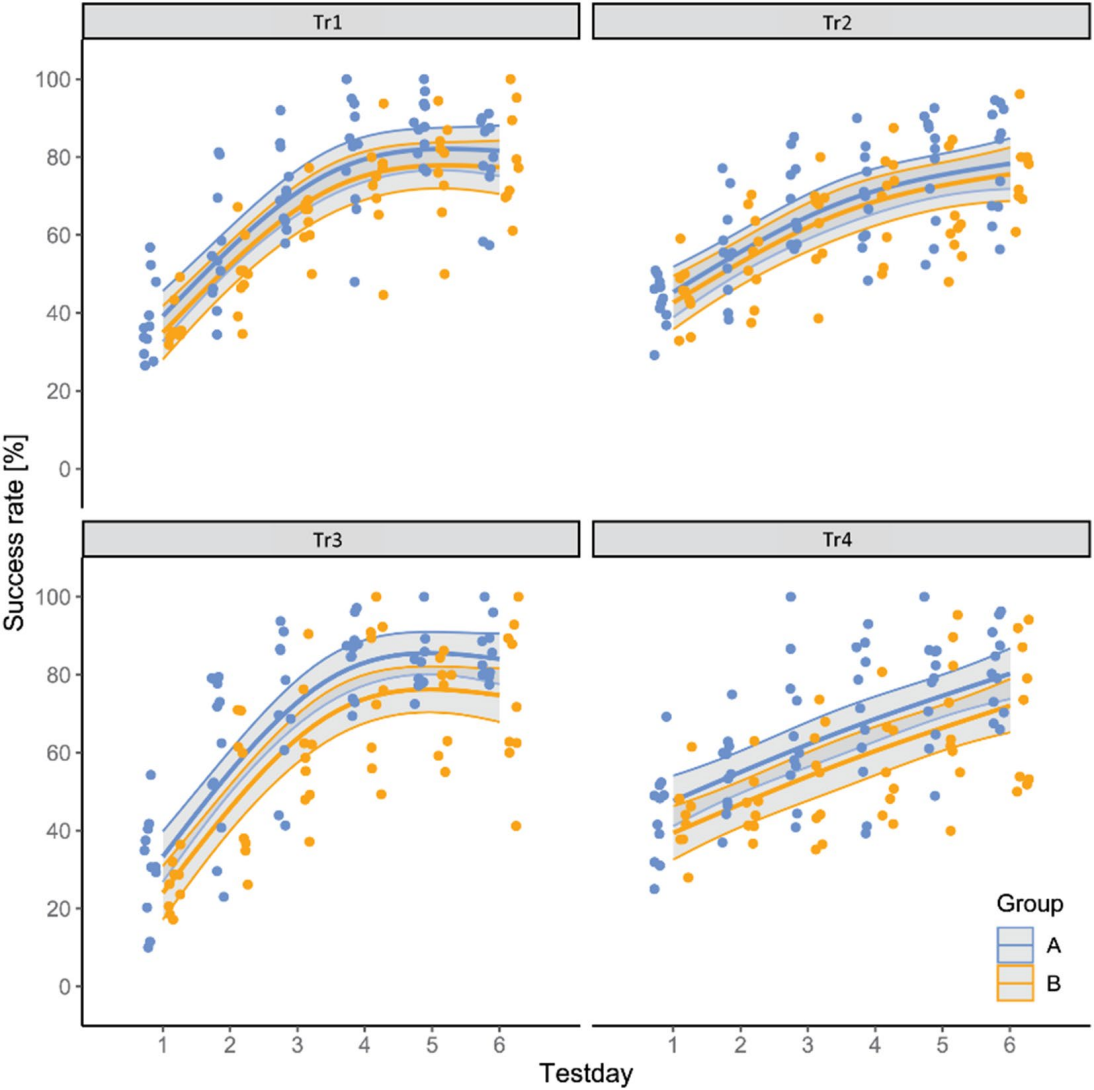
Estimated marginal means of individual success rates (%) along with 95% confidence intervals (grey) for Groups A (blue) and B (orange) across the six training days in the four training tasks (Tr1-Tr4)

Figure 4 shows the individual success rate estimates with 95% confidence intervals for Groups A and B across the six days of training in the four training tasks. The estimated marginal means (EMMs) were 76.1 (Group A) and 71.9 (Group B) for Tr1, 68.4 (A) and 65.7 (B) for Tr2, 79.3 (A) and 70.0 (B) for Tr3, and 65.5 (A) and 57.3 (B) for Tr4. We conducted an ANOVA (Table 2) to test for fixed effects. Significant effects on the success rate were found for *group* (*p* < 0.001) and the interaction *Tr* × *test day* (*p* < 0.001). Pairwise tests of the contrasts revealed that the success rate of Group A was higher than that of Group B in Tr3 (*p* < 0.011) and Tr4 (*p* < 0.024) [Table 2]. We calculated the effect size as Cohen’s d (Cohen 1988). We found a medium effect size for the contrast between the groups in Tr3 and Tr4 (see Table 2).

**Table 2:** ANOVA results based on the model fit of the success rate over the six training D.ys with chi-square statistics and P values of the fixed effects. Contrasts of the estimated marginal means between the groups were tested. The effect size is calculated as Cohen’s D

ANOVA table	Contrasts										
	Df	Chi sq	Pr (> Chi sq)	Task	Group	Estimate	SE	df	t ratio	*p* value	Cohen’s d
Tr	3	1.95	0.582	Tr1	A–B	4.23	3.61	285	1.17	0.243	0.327
Group	1	11.42	0.001	Tr2	A–B	2.72	3.61	283	0.753	0.452	0.211
Ns (Test day, 2)	2	465.70	<0001	Tr3	A–B	9.3	3.62	286	2.567	0.011	0.719
Tr: Group	3	2.26	0.519	Tr4	A–B	8.21	3.62	287	2.27	0.024	0.636
Tr: ns (Test day, 2)	6	42.67	<0.001								

**Fig. 5:**
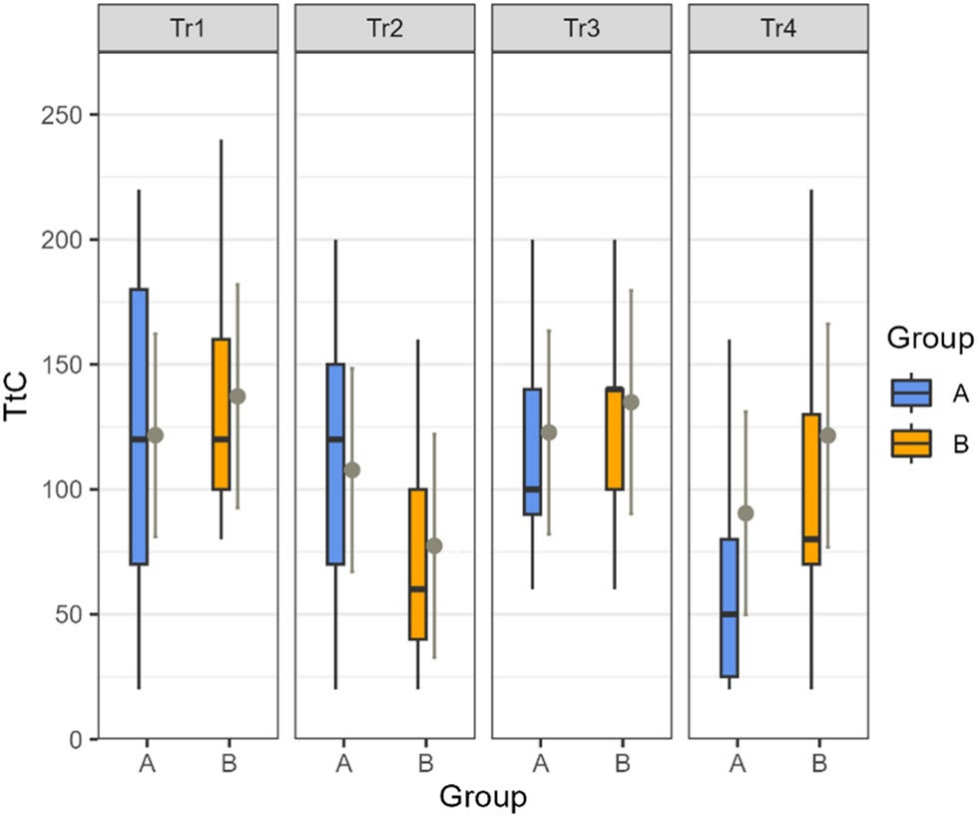
Number of trials needed by the animals in Groups A (blue) and B (orange) to reach the predefined learning criterion (TtC) in the four training tasks (Tr1-Tr4). The figure shows the raw data as box plots and the estimated marginal means plus the upper and lower confidence intervals

The number of trials the animals needed to reach the predefined learning criterion (TtC) is shown in Fig. 5. We did not find an impact of *group* or *Tr* or their interaction on the TtC. As seen from the p values of the LMM and the Tukey multiple comparison results, there was no significant difference in absolute learning performance (TTC) between the groups in any of the training tasks or between the training tasks. The animals needed between 77 (min, Group B, Tr2) and 137 trials (max, Group B, Tr1) to reach the TtC. As there are no significant difference in TtC between the groups in any of the training tasks, we did not calculate an effect sizes.

### Transfer test

The proportion of correct choices made by each animal for the first presentation of the new stimulus combinations (Tf1-Tf8) and the preferences for the correct photograph are shown in Table 3. In Group A, five of the 12 animals showed a preference for the correct photograph in the interspersed new stimulus combinations at the first presentation, and an additional two animals showed at least a trend towards this preference. Only one animal in Group B showed a preference for the correct photograph in the interspersed novel stimulus combinations.

**Table 3:** Percentage of correct first choices in the new stimulus combinations (Tf1-Tf8) interspersed in Tr4, tested via a binomial test (*P* = 0.25)

Group A			Group B		
Tier	% Correct	Binomial test	Tier	% Correct	Binomial test
1	42.9		29	33.3	
5	57.1	0.071	30	33.3	
7	85.7	0.001	31	50.0	
17	37.5		32	50.0	
26	42.9		33	16.7	
45	62.5	0.027	34	83.3	0.005
46	50.0		36	16.7	
47	28.6		42	33.3	
57	71.4	0.013	54	33.3	
62	57.1	0.071	64	50.0	
63	62.5	0.027	65	50.0	
76	71.4	0.013	74	50.0	

## Discussion

In a previous study (Langbein et al. 2023), based on visual discrimination training and a reversal test, we showed that the goats were not only able to discriminate between the photographs based on visual features alone, but also appeared to understand the relationship between the photographs and the conspecifics depicted. In addition, the results suggested that the goats had developed an awareness of the concept of familiarity (Zayan and Vauclair 1998; Stephan et al. 2013; Talbot et al. 2016).

In the present study two groups of goats were trained to perform identical visual discrimination tasks. The goats in group A were trained to select familiar individuals, whereas the goats in group B were trained to select unfamiliar individuals. Subsequent transfer test was conducted to assess their ability to generalise learned preferences to novel photographs of previously unseen goats. During the first training tasks (Tr1 and Tr2), no differences in learning performance between the two groups were observed. However, in the later tasks (Tr3 and Tr4), the goats in Group A exhibited better learning performance than did those in Group B. In the transfer test, five goats in Group A, but only one goat in Group B, demonstrated preferences for novel familiar or unfamiliar conspecifics. The superior performance of Group A goats in Tr3 and Tr4 and the number of goats that successfully transferred the familiarity concept to novel individuals provide compelling evidence for the formation of true image equivalence. In contrast, the worse performance of the goats in Group B underscores the greater cognitive complexity involved in recognising unfamiliar conspecifics as abstract categories.

Studies on nonhuman primates and various mammal and bird species have shown that photographs of familiar conspecifics are often favoured over pictures of unfamiliar individuals (Parr et al. 2000; da Costa et al. 2004; Racca et al. 2010; Coulon et al. 2011; but see Dawkins 1996; Ungerfeld et al. 2021). At the beginning of the present study, the goats lacked any prior experience or familiarity with interpreting photographs of conspecifics. The PT results suggest that only visual features of the photographs, which were probably perceived in the same way by the goats in both groups, led to the emergence of specific preferences. In other studies, confusion between objects and their real-life representations has been demonstrated for pictorially naive animals (Wright 1989; Parron et al. 2008). This parallels observations with humans; for example, individuals from cultures unfamiliar with photographic representations do not spontaneously perceive objects or persons in photographs but do so after the equivalence between the photograph and its reference has been explained or trained (Barley 1986; Deregowski 2001). Similarly, in Western societies, children typically begin to recognise images as symbolic representations of reality at approximately 2.5 years of age, following sufficient exposure to pictorial representations (DeLoache et al. 2003; DeLoache 2004). In the present study, at no point during the presentation of the photographs of conspecifics did we observe any reactions by the goats that would have indicated possible confusion between the images and real conspecifics.

What do we see in a picture showing, e.g., our uncle? We consider that the picture shows our uncle, as we have stored a mental representation of him as a single natural category (prototype) and assigned specific properties to this individual (Zayan 1994; Yorzinski 2017). However, what does a subject that has no experience with pictorial representations of real individuals see? What conclusions about social cognition can be drawn from goats discriminating photographic representations of conspecifics if we cannot determine how the animals “read” 2D images of familiar and unfamiliar conspecifics? When presented with photographic stimuli, many animal species rely on facial cues to distinguish between members of the same species of different sexes or ages. In the present study, there was no difference in learning performance in Tr1 and Tr2 between the groups, regardless of whether the animals had to learn to choose a familiar (Group A) or unfamiliar (Group B) conspecific. Both groups needed between 77 and 137 trials to satisfy the TtC. Their learning performance was comparable to that found in a recent study (Langbein et al. 2023) in which goats were tasked to discriminate photographs of conspecifics, as well as to the results of earlier studies in which goats were presented with simple or complex black symbols of the same size (Langbein et al. 2007a, 2008).

A major limitation of most related prior studies is that it remains unclear whether the animals involved also understand the equivalence between the depicted individual and the same subject in reality (Bovet and Vauclair 2000; Aust and Huber 2010). Ittelson (1996) defined three criteria that should be met before an animal can be assumed to understand the correspondence between the representation of an object and the real object. The first criterion is *perceptual correspondence*: The animal must be able to recognise that the representation perceptually corresponds to the actual object. The second criterion is *functional correspondence*: The animal must be able to use the representation in a functional way that reflects an understanding of its relationship to the real object. The third criterion is an *awareness of the representational status*: The animal must recognise that the representation is not the real object but rather represents or symbolises the real object. Similarly Fagot et al. (2001) proposed a conceptual framework with three levels of image processing. In the *independence mode*, which seems to represent the simplest cognitive level of processing pictures, images are discriminated based on salient visual features (patterns, configuration, colour, contrast, brightness, etc.). However, many experiments have not taken into account that discriminating between images of conspecifics is not equivalent to assuming that the images are perceived as representations of real individuals. In the second mode, called the *confusion mode*, the image is considered the functional and physical equivalent of the object it represents. In this mode of image processing, for example, dominant or submissive behaviour or even fear towards the image might be shown when images of conspecifics are presented, or an attempt may be made to grasp or eat the objects depicted. Such reactions have been observed in dogs, monkeys and primates (Rosenfeld and Vanhoesen 1979; Parron et al. 2008; Somppi et al. 2014). The mode of confusion can be seen as the starting point for the acquisition of image competence (DeLoache et al. 1998; Bovet and Vauclair 2000). Finally, in the *equivalence mode*, the subject shows representational insight, indicating an understanding of the relationship between the pictorial representation and the real object. This is accompanied by an awareness that images are entities that represent something other than themselves (DeLoache 2004).

Although various authors have long been sceptical about the broad evidence for true equivalence in the context of image processing by animals (Fagot et al. 2001; Bovet and Vauclair 2000), advanced experimental approaches such as reversal learning (Langbein et al. 2023; Suwandschieff et al. 2023), transfer learning (Coulon et al. 2011; Vonk and Povinelli 2011) and cross-modal recognition (Sliwa et al. 2011; Baciadonna et al. 2021) have demonstrated that, following sufficient experience with visual representations of objects, animals can understand the relationship between an object and its visual representation, thereby exhibiting representational insight (Aust and Huber 2010). Furthermore, numerous studies have revealed that both nonhuman primates (Parr et al. 2011; Talbot et al. 2015) and many other species exhibit preferences for photographs of familiar conspecifics over those of unfamiliar individuals (Kendrick et al. 1996; Coulon et al. 2009; Pitcher et al. 2017). In humans, this phenomenon has been described as the “familiarity effect” (Bruce et al. 2001). Dubois et al. (1999) reported that different areas of the human brain are activated when familiar and unfamiliar human faces are discriminated. In addition, in sheep, Kendrick et al. (2001) identified neurons in the temporal and medial prefrontal cortex that are specifically involved in the encoding of faces. Furthermore, for a subset of these cells, a preference for encoding the faces of familiar individuals within the animal’s current social environment was observed.

In the present study, the preference for familiar photographs emerged during training. In Tr1 and Tr2, no difference in learning performance was observed between the goats in Groups A and B; however, the animals in Group A demonstrated significantly faster learning in Tr3 and Tr4 than did those in Group B. The discrimination of pictures of familiar faces has been demonstrated to be superior to that of images of unfamiliar faces in humans (Ritchie et al. 2022), nonhuman primates (Adriaenssens and Johnsson 2011; Talbot et al. 2015), sheep (Kendrick et al. 1996; Peirce et al. 2001) and cattle (Coulon et al. 2011). This finding indicates that familiar faces are processed differently and with greater efficiency than unfamiliar faces or even those of other species. However, while the preference for pictures of familiar conspecifics over unfamiliar conspecifics suggests some level of understanding or recognition of the connection between 2D images and real individuals, it does not necessarily elucidate the relationship between the pictures and the actual individuals they represent. The animals may react only to the recognisable features that they have learned from direct experience with the individual and that they find in the images without fully understanding the abstract characteristics of pictorial representations as symbols or referential units (Aust and Huber 2006).

In the transfer test, five goats in Group A successfully applied the previously trained concept of familiarity to previously unseen goat pictures. Starting with the initial presentation of the interspersed novel stimulus combinations, these animals demonstrated significant preferences for photographs of familiar goats. Two other goats in Group A presented at least a corresponding tendency towards familiarity. These results suggest that the animals in Group A learned the concept of familiarity during training, which enabled them to categorise new photographs of members of their own group at their first presentation during the transfer tests. In contrast, only one animal in Group B demonstrated the capacity to transfer the trained concept of (un)familiarity to the interspersed novel stimulus combinations in the transfer test. The difference in performance in the transfer test between the animals in Groups A and B indicates that the concept of familiarity is more readily transferred to new photographs than is the concept of unfamiliarity. It is probable that the goats in Group A perform better because of repeated exposure to familiar conspecifics. Such repeated exposure leads to the formation of stronger mental representations, which in turn facilitates recognition across a variety of contexts, including photographs. Familiar individuals are likely to be more deeply encoded in memory, allowing faster and more accurate recognition, whereas more cognitive effort is needed to process and encode unfamiliar individuals, resulting in worse performance. This aligns with studies showing that individuals typically process and recognise familiar faces more efficiently than unfamiliar faces, leading to improved transfer performance across different contexts (Pokorny and de Waal 2009b).

The results of the present study show that goats can discriminate photographic images of conspecifics. At the beginning of training, they seem to be guided mainly by the visual features of the photographs. During the training, they begin to understand the connection between the photographs and the real conspecifics and the concept of familiarity. This process seems easier for animals that are trained to distinguish familiar from unfamiliar individuals than for animals trained to distinguish unfamiliar from familiar individuals. Thus, our results confirm that goats can learn real image equivalence, as suggested by our previous study (Langbein et al. 2023).

## Electronic supplementary material

Below is the link to the electronic supplementary material.

Supplementary Material 1

## Data Availability

The experimental data and the simulation results that support the findings of this study are available in Figshare with the identifier 10.6084/m9.figshare.27606921.
