# Supplementary figures and images for: Training and transfer test to study the referential understanding of conspecific photographs by goats
Source: Anim Cogn. 2025 Mar 13;28(1):22. doi: 10.1007/s10071-025-01945-2 (PMC11906514; doi:10.1007/s10071-025-01945-2)

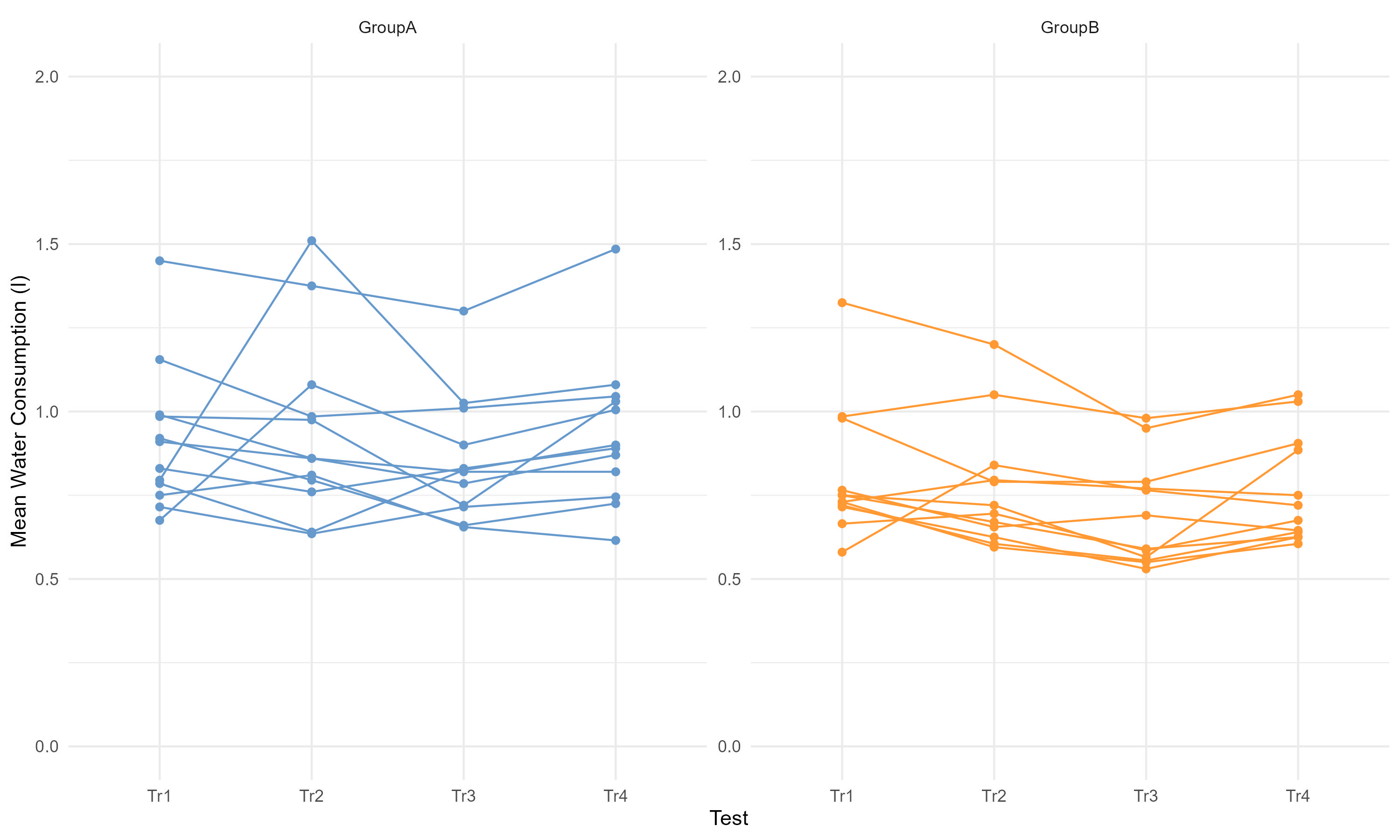

Supplement: Supplementary file 1 — Supplementary Material 1 [file 10071_2025_1945_MOESM1_ESM.jpg]
